# Polydiacetylene-coated polyvinylidene fluoride strip aptasensor for colorimetric detection of zinc(II)

**DOI:** 10.1016/j.snb.2016.03.118

**Published:** 2016-09

**Authors:** Jessica T. Wen, Karen Bohorquez, Hideaki Tsutsui

**Affiliations:** a Department of Bioengineering, University of California, Riverside, CA 92521, USA; b Department of Mechanical Engineering, University of California, Riverside, CA 92521, USA

**Keywords:** CR, color response, DA, diacetylene, DMPE, 1,2-dimyristoyl-sn-glycero-3-phosphoethanolamine, PDA, polydiacetylene, PVDF, polyvinylidene fluoride, RCS, red chromatic shift, RGB, red-green-blue, Polydiacetylene, Aptamer, Colorimetric, Zinc sensor, Polyvinylidene fluoride

## Abstract

We report a new polydiacetylene (PDA) sensor strip for simple visual detection of zinc ions in aqueous solution. The specificity of this sensor comes from Zn^2+^ DNA aptamer probes conjugated onto PDA. Effects of aptamer length and structure on the sensitivity of PDA’s color transition were first investigated. PDA conjugated with the optimal aptamer sequence was then coated onto a strip of polyvinylidene fluoride membrane and photopolymerized by UV exposure. The newly developed sensor successfully exhibited a blue-to-red chromatic change in a semi-quantitative manner in response to zinc ions. No discernable change was observed in solutions containing other common ions. Advantages of this sensor include its ease of fabrication, high specificity, and equipment-free detection, all of which are desirable for in-field applications and use in resource-limited settings.

## 1 Introduction

Polydiacetylene (PDA) materials have become popular in biosensing applications due to their unique optical properties that are readily discernable by the naked eye. PDA undergoes a chromatic change from blue to red in response to temperature [1], [2], [3], pH [4], [5], and molecular binding events [6], [7]. Furthermore, these chromatic changes can be made specific to the binding of a target analyte by conjugating detection probes onto PDA pendant side chains. In such systems, the detection probes can be antibodies [8], [9], [10], [11], proteins [10], [12], [13], or DNA aptamers [14], [15], [16]. Among them, DNA aptamers have several advantages, including but not limited to, ease of design, economical production, and chemical stability, all without compromising high specificity and affinity [17].

To date, analyte-specific PDA sensors are mostly developed in the form of a liposome (also referred to as a vesicle) in aqueous solutions [11], [18], [19] or a deposited layer on rigid substrate surfaces [20], [21], [22], [23]. However, due to complex sample handling requirements (e.g., multiple pipetting) and high costs of fabrication, these forms are not always ideal for biosensing applications in remote areas or resource-limited settings, which would most benefit from PDA’s instrument-free and naked-eye detection. Therefore, the development of PDA sensors in the form of a membrane strip, which are light, low cost, and easy to use, will be of significant benefit. Nevertheless, only a few PDA sensor strips have been reported and are limited to the detection of volatile organic compounds and solvents [24], [25], [26], [27], [28].

In this communication, we present the development of a new PDA sensor strip that is conjugated with DNA aptamers for the detection of Zn^2+^ in aqueous solutions. Other optical cationic sensors, such as traditional optodes, rely on spectrophotometric measurements of indicator dyes after their reaction with the target cation [29], [30]. The PDA sensor platform used in this study foregoes the need for bulky external analytical instruments and utilizes DNA aptamers for highly specific detection of Zn^2+^. First, using PDA liposomes, four candidate Zn^2+^ aptamers are compared to investigate effects of the aptamers’ hairpin structure, base length, and linker length. The aptamer design that induces chromatic changes most rapidly is then selected for the fabrication of a PDA sensor on polyvinylidene fluoride (PVDF) membrane. The sensor strip successfully undergoes a chromatic change discernable to the naked eye when dipped into Zn^2+^ solution, but not in solutions containing other ions.

## 2 Results and discussion

### 2.1 Effects of aptamer length, linker length, and structure on PDA liposome color transitions

Four variations, **1–4** (Table 1, Fig. S3), of a previously screened Zn^2+^ aptamer [31], were investigated for the effects of aptamer length and structure on the sensitivity of PDA color transitions. Aptamers **1** and **2** are hairpin aptamers composed of 65 nucleobases and differ by 6 carbons in the 5′ carbon linker between the first base and the liposome surface. Aptamers **3** and **4** are non-hairpin aptamers composed of 65 and 54 bases respectively. PDA liposomes composed of 1% (by total moles of lipids) of each Zn^2+^ aptamer were prepared (Supplementary Data, Section 3) and incubated in 500 μM Zn^2+^ solution for 30 m.

**Fig. 1:**
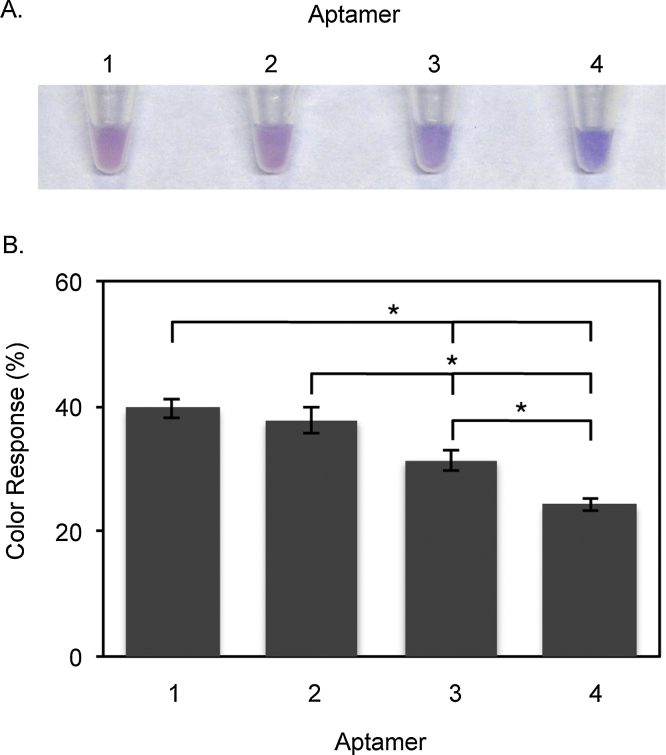
Color transitions of liposomes conjugated with four different aptamers (**1**–**4** in Table 1). (A) Images of liposome solutions after 30 m incubation in 500 μM Zn^2+^ solution. (B) Color response of liposomes after 30 m incubation in 500 μM Zn^2+^ solution. Color response data are represented as mean ± SD (n = 9). *p < 0.05. ​ (Colour version of this figure is available in the web version of this article.)

**Fig. 2:**
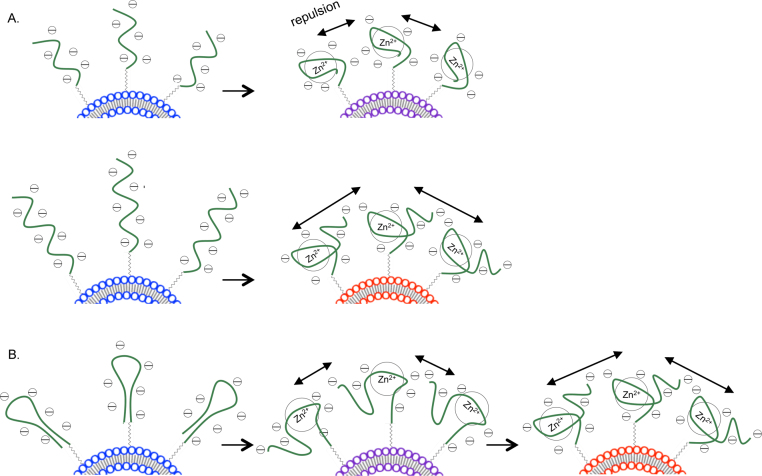
Schematic representation of Zn^2+^ detection by aptamers. (A) Aptamers of increasing length cause greater steric repulsion and lead to more sensitive color changes. (B) Un-folding of hairpin aptamers causes additional steric repulsion. (Colour version of this figure is available in the web version of this article.)

Color Response (CR) analysis [32] (Supplementary Data, Section 4) of liposomes conjugated with **1–4** after 30 m in Zn^2+^ solutions indicates that liposomes conjugated with **1** demonstrated the most significant color change (39.6% CR), followed by **2** (37.8% CR)**, 3** (31.4% CR) and **4** (24.2% CR) (Fig. 1). This suggests that an increasing aptamer length increases the sensitivity of color transitions. In particular, the difference in length by 11 bases between **3** (65 bases, non-hairpin) and **4** (54 bases, non-hairpin) resulted in a 7.2% CR difference. Contrastingly, **1** and **2** differ only by a length of 6 carbons in the carbon linker from the 5′ base to the original PDA side chain. This small change in the linker length has an insignificant effect on color transitions (Fig. 1B). While the mechanism behind PDA transitions remains to be fully understood, it is suggested that perturbations in the alternating −ene −yne backbone cause slight rotational changes that shift the optical absorption of the backbone from low (blue phase) to high energy (red phase) [33], [34], [35]. Specifically, the folding of aptamers conjugated to the surface of PDA liposomes around target biomolecules results in the formation of bulky aptamer-target groups which repulse one another. This steric repulsion at the liposomes surface disrupts the stabilizing hydrogen bonds between the PDA pendant side chains and translates into perturbations at the PDA backbone, which cause the liposome to change from blue to red [15], [16], [33]. Consequently, an increasing aptamer length results in even bulkier aptamer-target complexes and increased steric repulsion. This likely results in more sensitive color transitions (Fig. 2A).

Additionally, liposomes conjugated with **1** and **2** (hairpin aptamers), demonstrated a higher CR as compared to liposomes conjugated with **3** and **4** (non-hairpin aptamers). As hairpin aptamers, the structural conformation of **1** and **2** changes from a closed loop to an open loop upon binding with Zn^2+^ [31], [36]. As the aptamer unfolds to form the aptamer-target complex, the large conformational switch results in additional repulsion at the liposome surface (Fig. 2B). Additional studies are ongoing in our lab to further elucidate the mechanism by which the conformational switch of hairpin aptamers conjugated to the surface of PDA liposomes causes perturbations at the PDA backbone.

### 2.2 Testing of Zn^2+^ sensor strips

**Fig. 3:**
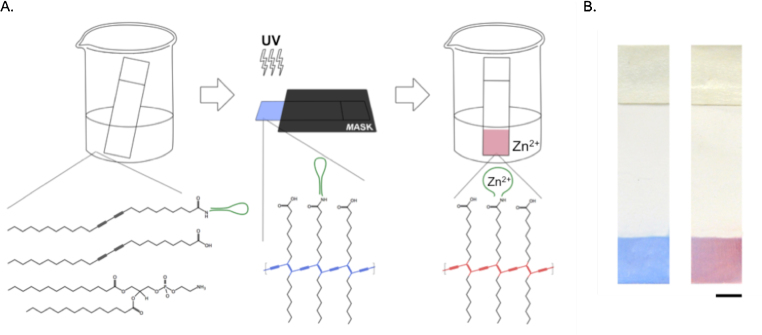
Preparation of PDA-coated PVDF strips. (A) PVDF strips are dipped into chloroform solution containing DA monomers conjugated with Zn^2+^ aptamer, unmodified DA monomers, and DMPE (left) and subsequently photopolymerized by 254 nm UV irradiation under a mask to yield a blue-colored device (middle). Dipping the device in Zn^2+^ solution causes a blue to pink/red color transition as a result of direct interaction between Zn^2+^ ions in solution and Zn^2+^ aptamers (right). (B) Prepared devices before (left) and after (right) color transition. Scale bar = 2.5 mm. (For interpretation of the references to colour in this figure legend, the reader is referred to the web version of this article.)

For the fabrication of our PDA sensor strip, **1** was selected as the detection probe due to the greatest CR reported by liposomes conjugated with it. To fabricate the sensor strip, PVDF strips were immersed in a chloroform solution containing 13.3% **1**-conjugated diacetylene (DA) monomers, 46.7% unmodified DA monomers and 40% DMPE phospholipid. PVDF has been previously employed in a fluorescence PDA sensor [37] and was selected as our sensor substrate due to its robust and inert properties that allow it to withstand harsh chemical environments and intense UV exposure [37], [38]. The addition of phospholipids in PDA sensors has been reported to enhance sensor signals without interrupting the chromatic properties of PDA [11], [39], [40], [41], [42]. Photopolymerization of the PDA layer with 254 nm UV light yielded a blue-colored area on the strip (Fig. 3).

**Fig. 4:**
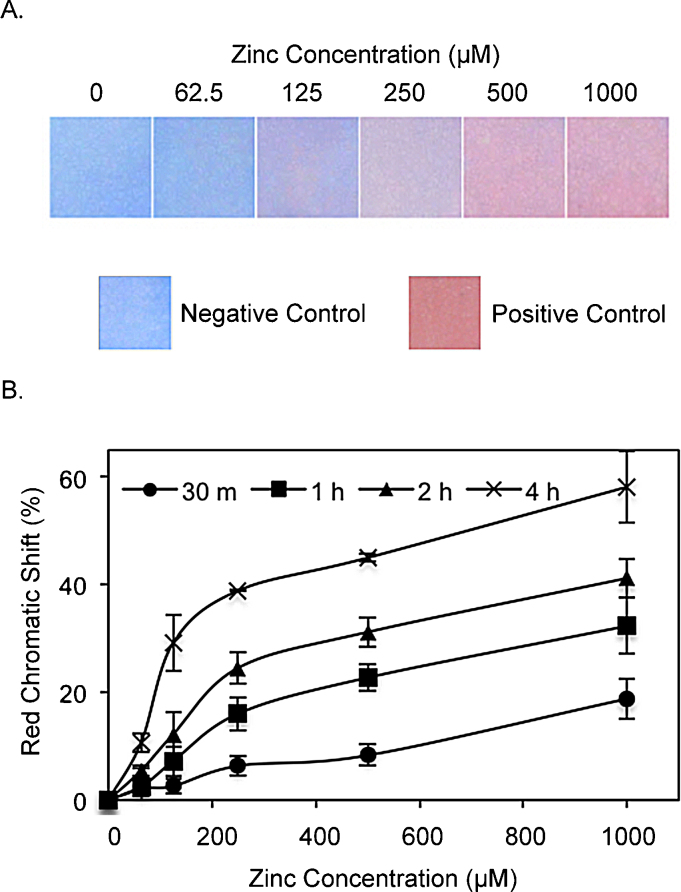
Zn^2+^ detection by PDA strips. (A) Images of the strips after 4 h incubation in Zn^2+^ solutions. The negative and positive control images are from strips dipped in deionized water and 1 M NaOH, respectively. (B) Red chromatic shift of the strips after 30 m, 1 h, 2 h and 4 h incubation in Zn^2+^ solutions. Red chromatic shift data are represented as mean ± SD (n = 3). (For interpretation of the references to colour in this figure legend, the reader is referred to the web version of this article.)

The PDA-coated PVDF strips (PDA strips) were dipped into solutions containing 0, 62.5, 125, 250, 500, and 1000 μM Zn^2+^ ions. The strips were imaged after incubation for 30 m, 1 h, 2 h and 4 h in the solutions. After 4 h in solution, they yielded a range of colors from blue to pink/red with increasing Zn^2+^concentration (Fig. 4A). For quantification of the chromatic transitions, color images were analyzed using ImageJ, an image processing software, to extract image-averaged red-green-blue (RGB) values. The RGB data were analyzed using digital colorimetric analysis [43], to generate a red chromatic shift (RCS) curve (Supplementary Data, Section 6) for each of the incubation periods indicated above (Fig. 4B). Previous studies have shown that a color shift of approximately 15% or greater is readily detectable by the naked eye [11], [44]. Accordingly, the limits of detection were determined to be 1000 μM (19% RCS) at 30 m, 500 μM (23% RCS) at 1 h, 250 μM (25% RCS) at 2 h, and 125 μM (29% RCS) at 4 h.

The RCS analysis of the PDA strips at 4 h indicates that two color transitions occurred. The first is at the detection limit of the sensor, 125 μM (8.16 ppm) Zn^2+^, at which a blue to purple color transition occurs. The second is at 500 μM (32.65 ppm) Zn^2+^ and above, at which the sensor yields a purple to pink/red color transition. This second transition corresponds to a 45% RCS, which is 16% higher than that of the purple transition at 125 μM (29% RCS), and is therefore readily discernable by the naked eye.

Zn^2+^ is an important nutrient found in staple food crops such as maize, rice, soybean, peanut, and cassava. Significantly, critical Zn^2+^ concentrations in these crops are in the range of 122–489 μM (8–30 ppm) (Table S2) [45], which align well with this sensor’s transitions. From an application standpoint, having more than one color transition is advantageous because each color can indicate a certain level of detection. Specifically, for the sensor described in this study, blue may serve to indicate Zn^2+^ deficiency (Zn^2+^ administration is needed), purple indicates Zn^2+^ levels in the critical range (no corrective action required), and pink/red indicates Zn^2+^ levels above the critical range (no more Zn^2+^ administration needed).

**Fig. 5:**
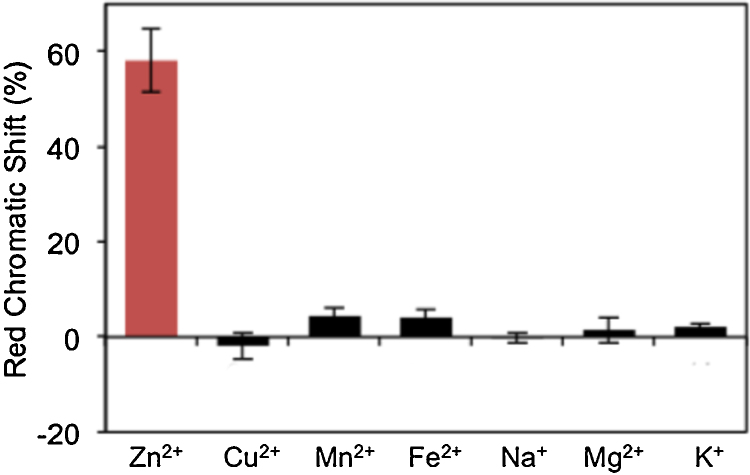
Red chromatic shift of the strips after 4 h incubation in solutions containing 1000 μM of various nutrients. All data are represented as mean ± SD (n = 3).

To test the sensor’s specificity, PDA strips were incubated in solutions containing 1000 μM of one of six nutrients Cu^2+^, Mn^2+^ and Fe^2+^, Na^+^, Mg^2+^ or K^+^ (as their chloride salts). RCS analysis of the strips after 4 h indicated that significant color transitions did not occur in any solutions containing ions other than Zn^2+^ (Fig. 5). Specifically, the RCS of the strips dipped in solutions containing Zn^2+^ is 13 times higher than that of strips dipped in solutions containing Mn^2+^, which displayed the highest chromatic shift from all of the control conditions. Notably, aptamer sequence **1** was previously reported to bind non-specifically to Cd^2+^ [31]. However, another biosensor employing **1** reported insignificant non-specific interactions with Cd^2+^ in control studies [46]. Because Cd is both highly toxic and carcinogenic, and is not one of the nutrients in food crops, this specific control was not included in the present study.

Finally to test the sensor’s stability, PDA strips were stored under multiple conditions and their sensitivity to Zn^2+^ was compared over the course of 28 days. Continuous exposure to a fluorescent tube light was shown to gradually lead to complete failure of the sensor (Fig. S9). On the other hand, storage of the strips in the dark had minimal effects on the sensor’s stability, and these strips were able to successfully detect Zn^2+^ after the 28 day period.

## 3 Conclusions

We observed that aptamer lengths and structural switches (such as those displayed by hairpin aptamers) significantly increase the sensitivity of color transitions in PDA liposomes. This provides insight into methods by which the color transitions of aptamer-conjugated PDA sensors can be optimized through changing the characteristics of conjugated aptamers. Subsequently, we demonstrated a PDA sensor strip for the discrimination of Zn^2+^ levels. Our sensor has a detection limit of 125 μM (8.16 ppm), which aligns well with the lower limit of critical concentrations in many food crops. Additionally, two distinct color transitions of the sensor enable three-stage, semi-quantitative detection. This technology can be easily adapted, by changing the detection probe (i.e., aptamer), to target a wide range of analytes from ions to pathogen biomarkers. Consequently, this sensor platform has a great potential for applications in many areas, including agriculture, environmental management and biomedicine.

## Figures and Tables

**Table 1:** Aptamer variations used in the study.

Aptamer	Sequence	5′ Carbon linker length	Number of bases	Hairpin
**1**	5′-GCATCAGTTAGTCATTACGCT TACGGCGGCTCTATCCTAACTGATATATTGTGAAGTCGTGTCCC-3′^a^	12	65	Yes
**2**	5′-GCATCAGTTAGTCATTACGCT TACGGCGGCTCTATCCTAACTGATATATTGTGAAGTCGTGTCCC-3′	6	65	Yes
**3**	5′-ATGCTGACCGATCATTACGCT TACGGCGGCTCTATCCTAACTGATATATTGTGAAGTCGTGTCCC-3′	12	65	No
**4**	5′-TCATTACGCTTACGGCGGCTC TATCCTAACTGATATATTGTGAAGTCGTGTCCC-3′	12	54	No

a Sequence from Ref. [31].
