# Supplementary material for: Polydiacetylene-coated polyvinylidene fluoride strip aptasensor for colorimetric detection of zinc(II)
Source: Sens Actuators B Chem. 2016 Sep;232:313–7. doi: 10.1016/j.snb.2016.03.118 (PMC4872522; doi:10.1016/j.snb.2016.03.118)
Supplement: Supplementary file 1 [file mmc1.docx]

**Supplementary Data**

**Polydiacetylene-coated polyvinylidene fluoride strip aptasensor for colorimetric detection of zinc(II)**

Jessica T. Wen,^a^ Karen Bohorquez^b^ and Hideaki Tsutsui^a,b*^

^a^Department of Bioengineering, University of California, Riverside, CA, 92521, USA

^b^Department of Mechanical Engineering, University of California, Riverside, CA, 92521, USA

**1. Materials**

The diacetylene monomer, 10,12-tricosadiynoic acid (TCDA), was purchased from GFS Chemicals (Powell, OH, USA). 1,2-dimyristoyl-sn-glycero-3-phosphoethanolamine (DMPE) was obtained from VWR International (Radnor, PA, USA). Organic solvents, polyvinylidene fluoride (PVDF) transfer membranes (EMD Millipore, 0.45 µm), and Slide-A-Lyzer MINI Dialysis Devices with molecular weight cut off of 3.5 kDa were purchased from Fisher Scientific (Pittsburgh, PA, USA). Amine-modified Zn^2+^ aptamers were purchased from Integrated DNA Technologies (Coralville, IA, USA). All other compounds used are research grade and were obtained from Sigma Aldrich (St. Louis, MO, USA).

**2. Preparation of NHS-modified diacetylene monomers (TCDA-NHS)**

Conversion of TCDA to a succinimide ester was carried out as previously described in literature [1, 2]. Briefly, 0.25 g (0.72 mmol) of TCDA, 0.26 g (1.35 mmol) of N-(3-dimethylaminopropyl)-N’-ethylcarbodiimide hydrochloride (EDC-HCl) and 0.12 g (1.07 mmol) of N-hydroxysuccinimide (NHS) were dissolved in 4 mL of methylene chloride. The solution was stirred with a magnetic stirrer for 2 h at room temperature. The solvent was evaporated by a stream of nitrogen and the residue was purified by extraction with ethyl acetate to yield TCDA-NHS monomers as a white solid; ^1^H NMR (400 MHz, chloroform-*d*) ∂ ppm 0.89 (t, 3 H) 1.26 - 1.79 (m, 28 H) 2.25 (t, 4 H) 2.61 (t, 2 H) 2.85 (s, 4 H) (Fig. S1).


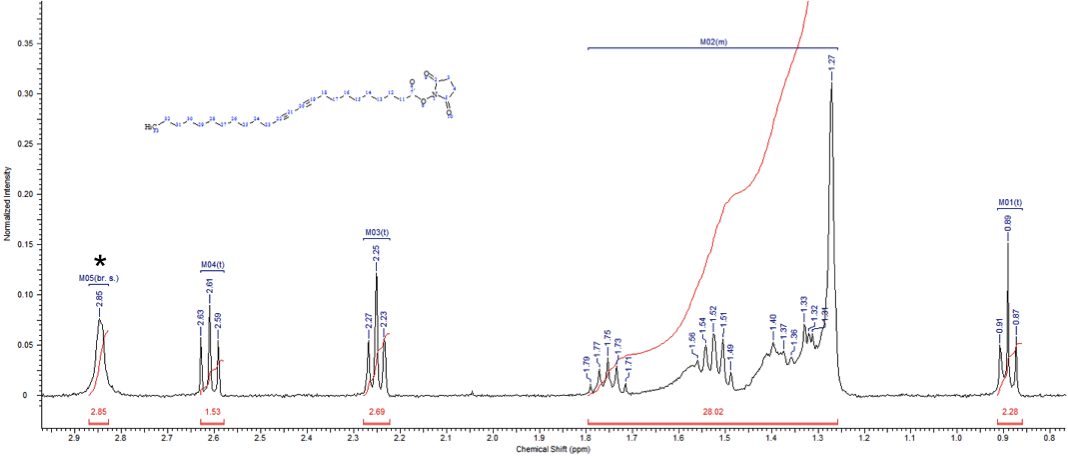


**Fig. S1.** TCDA-NHS NMR spectra, * indicates ^1^H peak from N-hydroxysuccinimide.

**3. Preparation of PDA liposomes for aptamer study**

25.5 nmol TCDA-NHS, 237 nmol TCDA and 487.5 nmol DMPE were mixed in chloroform, and the solvent was evaporated by a stream of nitrogen. The mixture was resuspended in deionized water to a total lipid concentration of 3 mM and sonicated (Qsonica Q500, 20% amplitude) at 80 ºC for 5 m. 7.5 nmol of one of four amine-modified Zn^2+^ aptamers (Table 1: **1-4**) was added to the solution and gently agitated (Fisher Scientific Mini-Tube Rotator) for 4 h to allow for the conjugation of the aptamers (Fig. S2, S3). Unreacted NHS was quenched by the addition of ethanolamine, and the solution was stored at 4 ºC overnight. Photopolymerization of the solution under 254 nm UV light (Spectroline E-series, 6 W) yielded a blue PDA liposome solution containing 1% TCDA-aptamer (by total moles of lipid). Dynamic light scattering (DLS, Malvern Zetasizer Nano ZS90) analysis indicated the mean diameter of the liposomes was approximately 120 nm (Fig. S4).


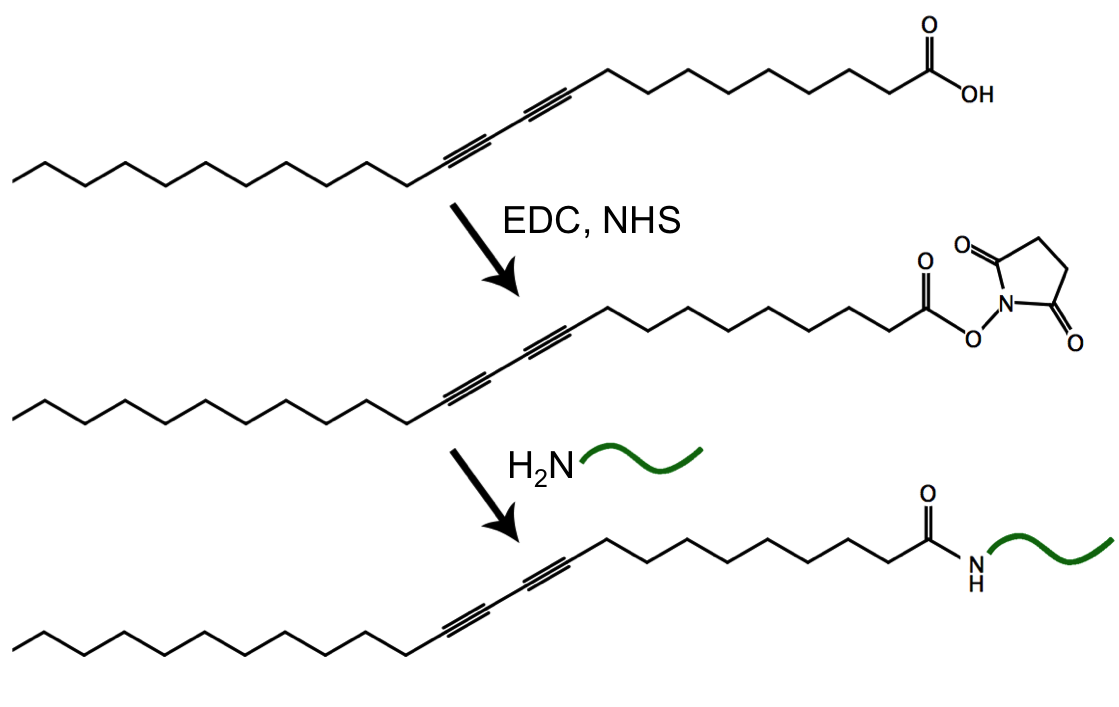


**Fig. S2.** Conjugation of aminated aptamer to diacetylenes through activation with N-(3-dimethylaminopropyl)-N’-ethylcarbodiimide (EDC) and N-hydroxysuccinimide (NHS).


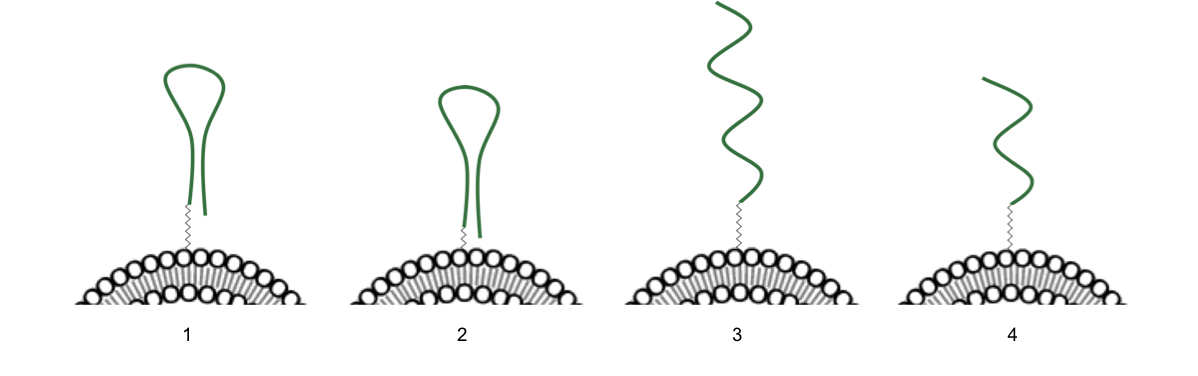


**Fig. S3.** Schematic depiction of aptamers **1-4** conjugated to the surface of liposomes. Image is not drawn to scale to exaggerate the differences between the aptamers.


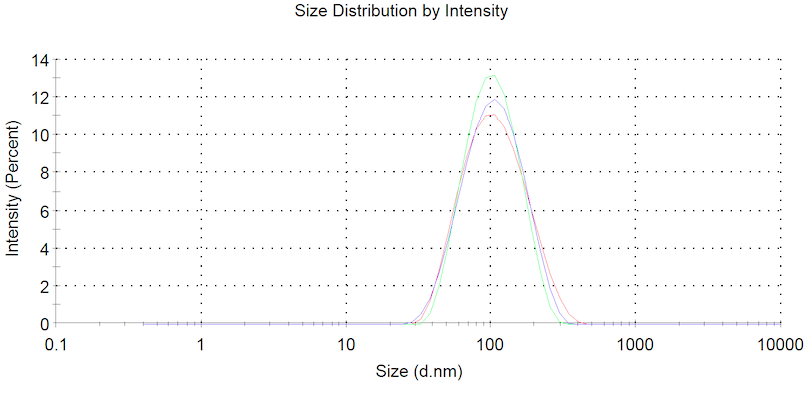
**Fig. S4.** DLS graph of PDA liposome size. All lines represent data recorded from a single sample.

**4. Analysis of PDA liposomes**

PDA liposomes were incubated with 5 mM Zn^2+^ for 30 m and absorption measurements were recorded by a spectrophotometer (Thermo Scientific NanoDrop 2000c) (Fig. S5). All experiments were performed in triplicates and three measurements were recorded for each sample. Color Response (CR) was calculated as previously described [3]. Briefly, the percent blue ($PB$), is first defined as:

$$PB=\frac{A_{blue}}{A_{blue}+A_{red}} \times100\%$$

where $A_{blue}$ is the absorbance at 647 nm (PDA blue form) and $A_{red}$ is the absorbance at 521 nm (PDA red form). Then, the CR is defined as:

$$CR=\frac{\left( {PB}_{0}-{PB}_{f} \right)}{{PB}_{0}} \times100\%$$

where ${PB}_{0}$ is the initial precent blue of untested liposomes and ${PB}_{f}$ is the final percent blue of liposomes incubated with Zn^2+^.


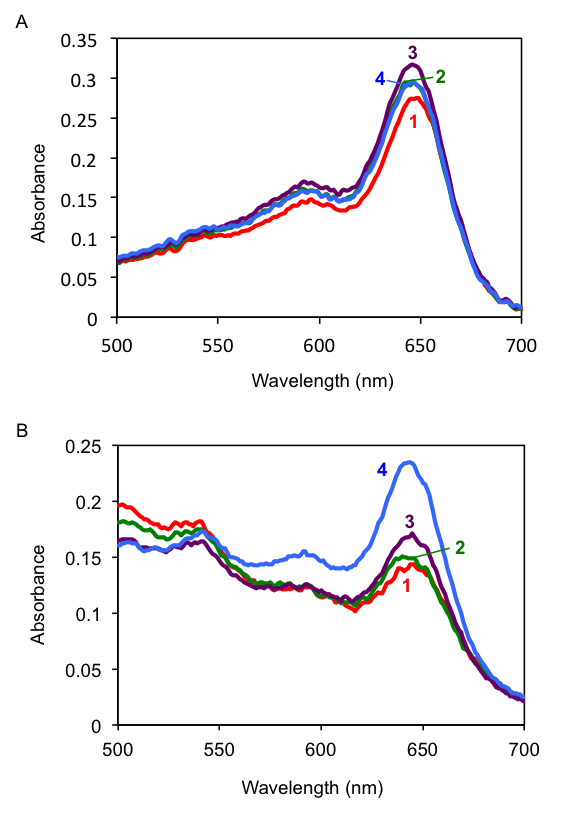


**Fig. S5.** Absorbance spectra of PDA liposomes conjugated with aptamers **1-4** before (A) and after (B) incubation in 5 mM Zn^2+^.

**
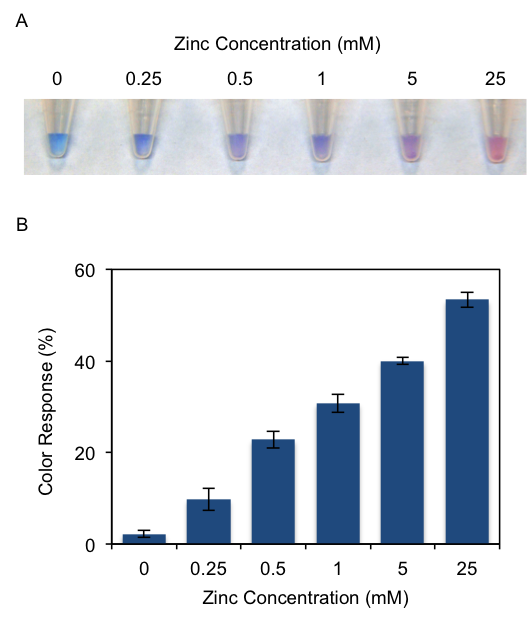
**

**Fig. S6.** Color transitions of liposomes with 1% of aptamer **1** after 1 h incubation in solutions containing 0 to 25 mM Zn^2+^. Color response data are represented as mean ± SD (n = 9).

**5. Preparation of PDA strip for zinc detection**

850 nmol of TCDA-NHS was incubated with 200 nmol of aptamer **1** in 300 µL methylene chloride for 4 h at room temperature in the dark. Unreacted compounds were removed by dialysis. A solution of TCDA-**1** (200 nmol), TCDA (700 nmol), and DMPE (600 nmol) was prepared in 500 µL chloroform. The total lipid concentration of the resulting solution was 3 mM with 13.3% TCDA-**1**. PVDF strips were prepared using a guillotine paper cutter and scissors. The strips were dipped into and immediately removed from the diacetylene solution and allowed to dry under ambient conditions in a fume hood (< 10 s). The strips were subsequently photopolymerized under a mask with 254 nm UV light for 75 s to yield blue-colored tips (Fig. 2).

**6. Colorimetric zinc detection and analysis of PDA strip sensor**

PDA-coated PVDF strips (PDA strips) were dipped into solutions containing various concentrations of Zn^2+^ (as ZnCl_2_) from 0 to 1 mM in deionized water at room temperature. All experiments were performed in triplicates. Color images of PDA strips were recorded using a Nikon D5100 digital camera at incubation times from 30 m to 4 h. Data from collected images were extracted using ImageJ software as 8-bit red-green-blue (RGB) values (Table S1). Red chromatic shift (RCS) was calculated using digital colorimetric analysis as previously described [4]. In short, the red chromaticity level ($r$), calculated as

$$r=\frac{R}{R+G+B}$$

depicts the relative intensity of the red component of the image. The RCS, which then defines the extent of the blue-to-red transition, is determined by:

$$\% RCS= \frac{r_{sample}-r_{0}}{r_{max}-r_{0}} \times100\%$$

where $r_{sample}$ is the average red chromaticity level of the sample image, $r_{0}$ is the average red chromaticity level of a baseline, and $r_{max}$ is the average red chromaticity level of a positive control image in which the maximum blue-to-red transition has occurred. $r_{0}$ and $r_{max}$ were determined using PDA strips dipped in deionized water and 1 M NaOH, respectively. 1 M NaOH solution is routinely used as a positive control because its high pH (=14) readily induces clear red transitions [4].

To extract further information from the RGB data set (Table S1, Fig. S7), principal component analysis (PCA) of three variables (red, green, blue) was employed using the Microsoft Excel add-in Multibase package (Numerical Dynamic, Japan). PCA is a mathematical transformation of multivariate data that is often used to statistically visualize RGB data sets due to their high dimensionality, where each image is represented by three RGB values [5-7]. The transformation reduces dimensionality of the RGB data, converting the data set to a new PCA score plot, on which the axes are determined by orthogonal factors drawn from the data that maximize the variance of the data set [8, 9]. The first principal component (PC1) is expressed as PC1 = 0.71 x R + 0.00 x G – 0.71 x B, and the second principal component (PC2) as PC2 = – 0.19 x R – 0.96 x G – 0.19 x B. The 2D PCA score plot indicates that PC1 accounts for 63.8% of the total data variance and PC2 accounts for 35.9% (Fig. S8). Distinctly, the PCA plot can be divided into three clusters. The first cluster contains RGB values from 0 and 62.5 µM Zn^2+^, the second cluster contains values from 125 and 250 µM Zn^2+^, and the third cluster contains values from 500 µM and 1000 µM Zn^2+^. This further confirms three colors exhibited by the sensor, blue, purple, and pink/red, which were also indicated by the RCS analysis (Fig. 4A, 4B).

**Table S1.** RGB values extracted from strips after 4 h in Zn^2+^ solution

| **Sample** | **Red** | **Green** | **Blue** |
| --- | --- | --- | --- |
| 0 µM - 1 | 143.8 | 182.2 | 240.4 |
| 0 µM - 2 | 104.3 | 154.0 | 243.1 |
| 0 µM - 3 | 113.5 | 161.1 | 244.3 |
| 62.5 µM - 1 | 143.2 | 180.6 | 238.8 |
| 62.5 µM - 2 | 150.4 | 186.3 | 240.4 |
| 62.5 µM - 3 | 135.9 | 164.9 | 233.7 |
| 125 µM - 1 | 173.3 | 179.6 | 223.7 |
| 125 µM - 2 | 169.4 | 172.6 | 220.1 |
| 125 µM - 3 | 163.1 | 185.4 | 229.6 |
| 250 µM - 1 | 177.1 | 169.9 | 216.2 |
| 250 µM - 2 | 187.2 | 184.2 | 222.9 |
| 250 µM - 3 | 179.2 | 175.9 | 214.4 |
| 500 µM - 1 | 179.5 | 164.8 | 206.2 |
| 500 µM - 2 | 187.8 | 172.6 | 210.8 |
| 500 µM - 3 | 184.4 | 170.9 | 208.7 |
| 1000 µM - 1 | 197.5 | 176.1 | 209.4 |
| 1000 µM - 2 | 204.0 | 162.4 | 198.4 |
| 1000 µM - 3 | 205.6 | 164.7 | 195.6 |


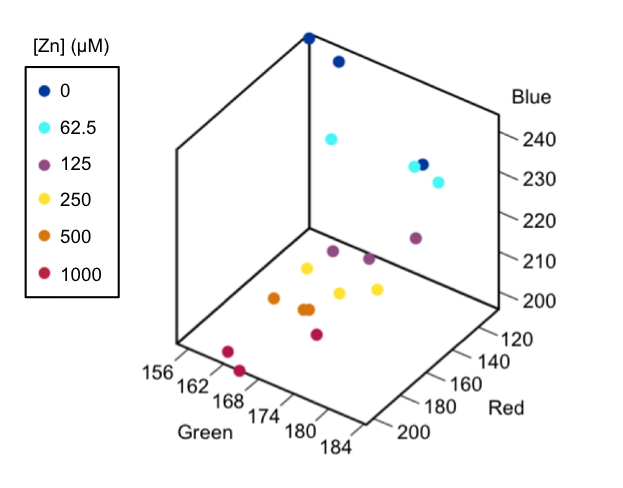


**Fig. S7.** 3D plot of RGB values.


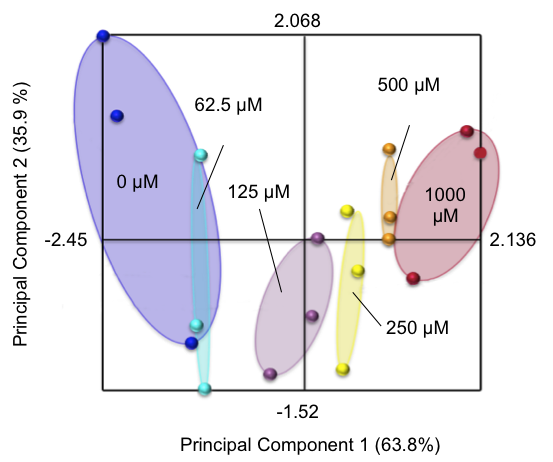


**Fig. S8.** PCA transformation of RGB values.

The stability of the PDA strips was evaluated over a 28-day period during which strips were stored under four different conditions and tested every 7 days (Fig. S9). The storage conditions included (1) under normal air, at room temperature, in the dark, (2) under normal air, at 4 ºC, in the dark, (3) under N_2_ gas, at room temperature, in the dark, and (4) under normal air, at room termperature, under constant light. RCS analysis shows that all strips stored in the dark demonstrated successful Zn^2+^ detection after 28 days (Fig. S9B, S9C). Strips stored under constant light exhibited non-specific color transition that progressed over time and were not sensitive to Zn^2+^ solutions at the end of the 28-day period. A successful strip was determined as one that demonstrated a blue to pink/red transition that is readily discernable by the naked eye after 4 h incubation in 1000 µM Zn^2+^ solution.


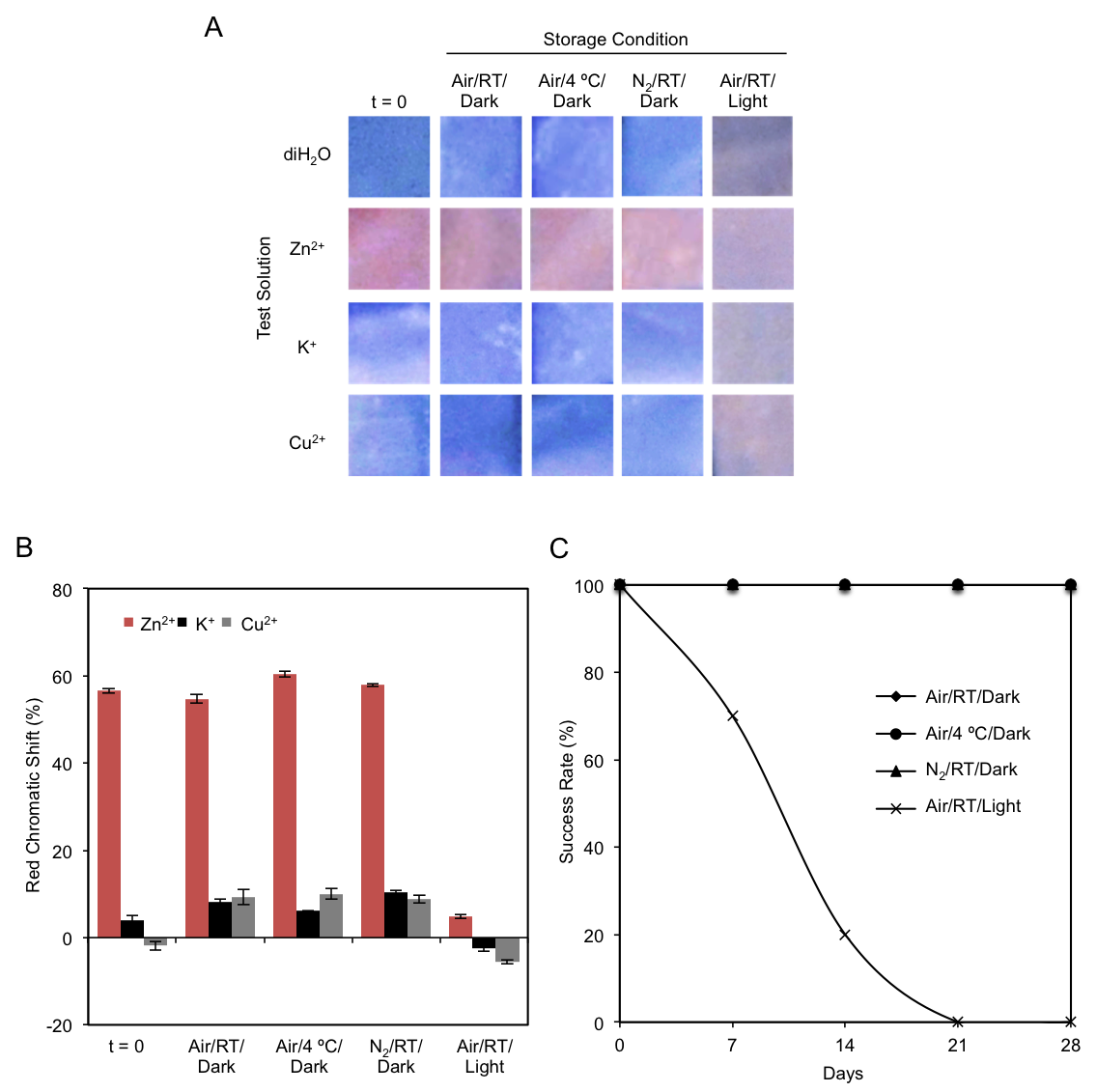


**Fig S9.** Stability test of PDA strips over 28 days. (A) Images of tested strips immediately after production (t = 0) and after storage under various conditions for 28 days. Strips were imaged after 4 h incubation in deionized water or 1000 µM solutions containing Zn^2+^, K^+^ or Cu^2+^. (B) Red chromatic shift of tested strips after 28 days of storage under various conditions. Data are represented as mean ± SD (n = 10 for strips tested in Zn^2+^ solutions, n = 3 for strips tested in control solutions). (C) Success rate of strips under various conditions over a 28-day period. Success rates were calculated as a percentage of successful strips out of 10 for each storage condition. RT = room temperature.

**7. Zinc levels in food crops**

**Table S2.** Critical Zn^2+^ Concentrations*

| **Crop** | **Tissue** | **Critical [Zn] (ppm)** |
| --- | --- | --- |
| maize | upper third leaves | 15-16 |
| rice | whole shoot | 10-20 |
| soybean | upper recently matured leaves, early bloom | 20 |
| peanut | youngest fully emerged leaf early pegging | 8-10 |
| wheat | shoot | 32 |
| canola | shoot | 23 |
| sorghum | youngest fully emerged leaf | 8-10 |
| cassava | youngest mature blade | 30 |

*as reported by [10]

**References**

[1] J. Lee, H. Jun, J. Kim, Polydiacetylene–liposome microarrays for selective and sensitive mercury(II) detection, Adv. Mater. 21 (2009) 3674-3647.

[2] J. Lee, H.-J. Kim, J. Kim, Polydiacetylene liposome arrays for selective potassium detection, J. Am. Chem. Soc. 130 (2008) 5010-5011.

[3] S. Okada, S. Peng, W. Spevak, D. Charych, Color and chromism of polydiacetylene vesicles, Accounts Chem. Res. 31 (1998) 229-239.

[4] R. Volinsky, M. Kliger, T. Sheynis, S. Kolusheva, R. Jelinek, Glass-supported lipid/polydiacetylene films for colour sensing of membrane-active compounds, Biosens. Bioelectron. 22 (2007) 3247-3251.

[5] Y. Li, L. Wang, X. Yin, B. Ding, G. Sun, T. Ke, et al., Colorimetric strips for visual lead ion recognition utilizing polydiacetylenes embedded nanofibers, J. Mater. Chem. A, 2 (2014) 18304-18312.

[6] T. Soga, Y. Jimbo, K. Suzuki, D. Citterio, Inkjet-printed paper-based colorimetric sensor array for the discrimination of volatile primary amines, Anal. Chem. 85 (2013) 8973-8978.

[7] B. Yoon, I.S. Park, H. Shin, H.J. Park, C.W. Lee, J.M. Kim, A litmus‐type colorimetric and fluorometric volatile organic compound sensor based on inkjet‐printed polydiacetylenes on paper substrates, Macromol. Rapid Comm. 34 (2013) 731-735.

[8] S. Wold, K. Esbensen, P. Geladi, Principal component analysis, Chemom. Intell. Lab. Syst. 2 (1987) 37-52.

[9] M. Ringnér, What is principal component analysis?, Nat. Biotechnol. 26 (2008) 303-304.

[10] B.J. Alloway, Zinc in soils and crop nutrition, Brussels, Belgium: International Zinc Association 2004.
